# Moral uncertainty and the farming of human-pig chimeras

**DOI:** 10.1136/medethics-2018-105227

**Published:** 2019-06-29

**Authors:** Julian Koplin, Dominic Wilkinson

**Affiliations:** 1 Murdoch Children’s Research Institute, Parkville, Victoria, Australia; 2 University of Melbourne Law School, Carlton, Victoria, Australia; 3 Oxford Uehiro Centre for Practical Ethics, Faculty of Philosophy, University of Oxford, UK; 4 John Radcliffe Hospital, Oxford, UK

**Keywords:** moral status, chimeras, stem cell research, transplantation

## Abstract

It may soon be possible to generate human organs inside of human-pig chimeras via a process called interspecies blastocyst complementation. This paper discusses what arguably the central ethical concern is raised by this potential source of transplantable organs: that farming human-pig chimeras for their organs risks perpetrating a serious moral wrong because the moral status of human-pig chimeras is uncertain, and potentially significant. Those who raise this concern usually take it to be unique to the creation of chimeric animals with ‘humanised’ brains. In this paper, we show how that the same style of argument can be used to critique current uses of non-chimeric pigs in agriculture. This reveals an important tension between two common moral views: that farming human-pig chimeras for their organs is ethically concerning, and that farming non-chimeric pigs for food or research is ethically benign. At least one of these views stands in need of revision.

It may soon be possible to grow human organs inside human-pig chimeras via a technique known as interspecies blastocyst complementation. This technique involves two key processes. First, the genes responsible for growing a specific organ are knocked out of a pig embryo (eg, using CRISPR-Cas9 genome editing). Second, human pluripotent stem cells are injected into the pig embryo, which is then implanted into a sow and allowed to develop. The technique, if successful, should create a part-human chimera—a creature comprising cells from different embryonic origins. Most of the chimera’s organs and tissues would be composed of a mix of (mainly) pig and (some) human cells. However, some specifically targeted organs would be wholly human. Because the pig embryo has been genetically altered to prevent the growth of one or more specific organs, the human stem cells should fill this ‘developmental niche’ to grow functional human organs inside the chimeric pig’s body.^1^

Organ generation via interspecies blastocyst complementation is on the cusp of feasibility. Interspecies blastocyst complementation has already been used to generate rat organs inside rat-mouse chimeras^2 3^ and, conversely, to generate mouse organs inside mouse-rat chimeras.^4^ Scientists have even achieved some success between humans and other large mammals. A study recently published in *Cell* has described the creation of human-pig chimeric fetuses, which were created by introducing human stem cells to pig embryos, implanting them into a sow and allowing them to develop for 4 weeks. By the end of this process, human cells could be found throughout multiple tissues of the fetus. Although significant practical obstacles remain before human organs could be grown inside chimeric pigs, the authors claim their success ‘raise[s] the possibility of xeno-generating transplantable human tissues and organs towards addressing the worldwide shortage of organ donors.’^5^ Not only could this technique help provide organs to those who would not otherwise receive them, it might also circumvent the need for lifelong immunosuppression. This is because organs could theoretically be generated using stem cells derived from the patients themselves, in which case the organs would be a genetic match for their intended recipient.^6^

This paper discusses what is arguably the central ethical concern raised by the prospect of generating human organs in human-pig chimeras: that human-pig chimeras would develop morally relevant cognitive capacities.^i^ This concern has played a central role in both the broader bioethical discussions of human-animal chimeras^7 8^ and in recent public debates surrounding interspecies blastocyst complementation.^9^ For example, Lori Marino—writing in *Stat*—argued against creating human-pig chimeras due to the risk that such chimeras could develop highly sophisticated cognitive capacities:

These organisms may be capable of self-awareness to the extent that they understand their identity and circumstances, which would produce unbearable suffering… If we cannot say with certainty that this will never happen, then we need to stop this kind of research right now before we find ourselves in a world where there is no line.^10^

Paul Knoepfler—writing in *Wired*—argued that if human cells contribute to human-pig chimeras’ brains, the resulting creatures will occupy an ethical ‘grey zone’ between the moral status of animals and humans. Although Knoepfler suggested these concerns could theoretically be mitigated by limiting human cells’ contribution to chimeric animals’ brains, he argued that an appropriate threshold will be extremely difficult to define:

There’s no clear dividing line on the question of ‘overly’ human chimeric brains because we lack an understanding of at what point ‘humanization’ of an animal brain could lead to more human-like thought or consciousness. We don’t even know when this happens in the normal developing human brain.^11^

Julian Savulescu has argued that human-pig chimeras should be treated according to the highest level of moral status they could plausibly possess. This would require us to treat human-pig chimeras as if they have high moral status until they are proven to lack cognitive abilities beyond those of regular, non-chimeric pigs:

In the absence of conclusive research on these questions, any such chimera should be accorded the highest moral status consistent with its likely nature…. [I]f it could plausibly have higher cognitive functions, it should be treated as if it would have them. In considering the new life forms we create, we should err on the side of sympathy and generosity.^12^

Policy discussions of human-animal chimeras (including, but not limited to, chimeras created via blastocyst complementation) are likewise dominated by the concern that animals with chimeric brains might develop human-like cognitive capacities.^8^ Groups including the UK Academy of Medical Sciences,^13^ Japanese Expert Panel on Bioethics^14^ and German Ethics Council^15^ have recommended that research involving chimeric animals with humanised brains should be subject to greater restrictions than other forms of chimera research. In the USA, the National Institutes of Health has issued a moratorium on funding for blastocyst complementation as it considers ethical issues raised by the prospect of human cells contributing to chimeric animals’ brains.^16^ Concerns about chimeric animals’ moral status are most commonly and most forcefully raised regarding brain chimera research involving non-human primates^17 18^ or other large mammals,^13^ although some argue that we should not create *any* chimeras with human-like neuronal structures, regardless of species, due to concerns that such animals would develop human-like consciousness.^19^ Although there is limited bioethical work specifically on the creation of human-pig chimeras via blastocyst complementation, the literature that does exist often recommends creating safeguards against the humanisation of chimeric animals’ brains to ensure these creatures do not develop human-like thought or consciousness.^1 20 21^

This paper highlights an important tension between this trepidation regarding the farming of human-pig chimeras as a source of transplantable organs and the current widespread acceptance of the farming of non-chimeric pigs as a source of food. We argue below that ethical concerns about the creation of human-pig chimeras with partly humanised brains are best understood in terms of the uncertain moral status of these chimeric animals. We further show that the same factors that render uncertain the moral status of human-pig chimeras *also* render uncertain the moral status of regular, non-chimeric pigs. There is therefore an important inconsistency between many people’s attitudes towards the farming of human-pig chimeras and the farming of non-chimeric pigs. Either the creation of human-pig chimeras via blastocyst complementation is less morally concerning than is commonly assumed, or the use of non-chimeric pigs in livestock is more morally concerning than is commonly assumed—or possibly both.

First, however, it is worth clarifying what we mean when we discuss the moral status of human-pig chimeras and regular, non-chimeric pigs. To say that a being has moral status is to say that a being has moral importance in their own right; beings with moral status have interests that ought to be taken into account when we consider how we should treat them. We assume that moral status can be ascribed in degrees. To say that one being has *more* moral status than another is to say that our moral obligations towards the first being are weightier than our moral obligations towards the second. Depending on one’s philosophical commitments, moral status might be thought to vary because some kinds of beings deserve greater moral consideration than others, or it might vary because of differences in the nature and strength of different kinds of beings' interests.^22^ We believe the kinds of concerns about human-pig chimera research discussed above are most naturally expressed as concerns about chimeric animals’ degree of moral status. The concern seems to be that human-pig chimeras could develop characteristics that increase their moral status beyond that of a regular pig, potentially even to the point that their moral status approaches that of a regular human adult.

The arguments of this paper are also compatible with the view that full moral status is a threshold concept—that is, that it is a feature of beings who have at least a threshold level of some capacity or set of capacities.^23^ For those who view moral status this way, the concern about human-pig chimeras is that there is a non- probability that some of these animals might cross the threshold to full moral status.

We would also like to assuage a possible concern about this paper’s motivation. It might seem unusual, or even flippant, for an analysis of the ethics of chimera research to segue into a discussion of the ethics of eating meat. Unusualness aside, we think our argument has important practical implications. If our argument is correct, we either need to become more accepting of (and impose fewer restrictions on) the creation of part-human chimeras with humanised brains, or else become less accepting of (and impose greater restrictions on) the farming of non-human animals for food. Either approach will require us to rethink important human practices.

We also have a second aim that is more concretely tied to current bioethical controversies regarding part-human chimeras. By highlighting a problem with current ethical thought regarding human-pig chimeras, we hope to prevent this problem from slipping unnoticed into (and potentially distorting) future ethical analysis of part-human chimera research. Our second aim, in other words, is to help clear the intellectual space required to untangle the ethical issues raised by part-human chimeras.

## Moral uncertainty regarding human-pig chimeras

It is widely believed that our moral obligations towards non-human animals depend partly on their cognitive abilities, with the interests of humans privileged over those of non-human animals because humans typically possess some unique set of morally relevant cognitive capacities. On this view, if a part-human chimera acquires whatever set of cognitive capacities undergird humans’ moral status, it would also acquire the same degree of moral status we usually afford humans. It would be gravely wrong to treat humans in many of the ways we currently treat animal research subjects, or to raise and slaughter humans for their organs. By extension, it would be gravely wrong to use human-pig chimeras in research, or to use them as a source of transplantable organs, if these chimeric animals acquire cognitive capacities that confer human-like moral status.

The moral permissibility of generating human organs inside of human-pig chimeras therefore turns, in large part, on the moral status of these chimeric animals. The problem is that determining a human-pig chimera’s moral status would be very difficult. The first difficulty is philosophical. In order to accurately determine the moral status of a human-pig chimera we would first need to know precisely what capacities confer what degree of moral status. But this, of course, remains a major unresolved question in moral philosophy. The second difficulty is epistemological. Once we determine what capacities confer what degree of moral status, we will need to devise some way of testing for these capacities in chimeric animals that look, behave and communicate very differently to humans. There are many reasons why this would be difficult to achieve, including because we cannot speak with human-pig chimeras directly, because human-pig chimeras’ cognition would likely be influenced by their embodiment in porcine bodies and the conditions under which they are raised, and because inferences drawn from animal behaviour or physiology are often open to question.^24^ The moral status of human-pig chimeras is therefore doubly uncertain. We would be uncertain of what cognitive capacities such chimeras possess, and we would further be uncertain of what set of capacities are relevant to its moral status.

Perhaps this conclusion is too quick. It is sometimes argued that even if chimeric animals have the latent ability to develop morally relevant cognitive abilities, the animal would need to be raised under the ‘right’ set of social conditions for these abilities to develop. Given that laboratory conditions are unlikely to provide the necessary forms of interaction and socialisation (and given, moreover, that human-pig chimeras could be killed while they are still quite young), we should not be overly concerned about chimeric animals’ moral status.^24 25^ Or so the argument goes.

To see where this argument goes wrong, consider the following thought experiment:

*Infant organ harvesting*: It is 2040. The problem of shortage of kidneys for transplantation has been solved. Patients who have kidney failure have stem cells harvested. Those cells are then inoculated into a human fetus that has been modified via CRISPR technology to knock out the genes that normally drive development of that organ. The fetus is gestated in a surrogate, born alive and then euthanised at a few minutes of age.

Presumably, virtually no one would accept infant organ harvesting. While the infant’s cognitive capacities are not those of a fully grown human, they would develop further if they had not been killed. Killing the infant before they have a chance to manifest or develop more sophisticated self-awareness, communication or relationships would not mitigate or remove the wrongness of killing them. If these factors do not mitigate the wrongness of killing an infant, they cannot mitigate the wrongness of killing human-pig chimeras who also would have gone on to develop morally relevant cognitive capacities.

What should we do if we are uncertain about the degree of moral status possessed by human-pig chimeras? We would make a grave moral mistake if human-pig chimeras have a high degree of moral status and we mistakenly treat them as if they have only the lower degree of moral status that is commonly attributed to non-chimeric pigs. In particular, if human-pig chimeras possess a degree of moral status that is equivalent (or very close) to that of a normal human, then killing them for their organs would be morally equivalent (or very close to equivalent) to infant organ harvesting. We should therefore be confident that human-pig chimeras would lack morally relevant cognitive capacities before setting out to use them as a source of transplantable human organs.^ii^

This leaves open the question of *how* confident we should be before setting out to use human-pig chimeras a source of transplantable organs. One way of unpacking this question would be to imagine that there is some probability X that the chimeric animals have cognitive capacities that are equal to cognitively normal humans, and that the wrongness of killing them would be equivalent to the killing of a child to harvest their organs. The question, then, is what value of X would be sufficiently low for generating chimeras to be acceptable?

Here is another thought experiment:

*Low probability infant organ harvesting*: It is 2060. The problem of shortage of kidneys for transplantation has been solved. Patients who have kidney failure have stem cells harvested. Those cells are then inoculated into a pig fetus. This is highly likely to generate a viable organ for transplantation, and research has been done that unequivocally shows that the chimeric pigs have no human neural tissue. However, in X% of cases, no viable pig organ is able to be generated. In such a situation, a human fetus is used instead. The human fetus is gestated in a surrogate, born alive and then euthanased at a few minutes of age.

Those who hold strong deontological views might hold that farming human-pig chimeras would be morally impermissible unless X is zero. A consequentialist might set the threshold for X somewhat higher, given that the harms to human infants would need to be balanced against the potential benefits we might achieve by generating organs in this way. Exactly where a consequentialist should set X is, of course, an open question. In general, however, given that most people would find it highly unethical to harvest organs from normal human children, most people would presumably set the value of X quite low.

The above argument should be relatively uncontroversial. Indeed, something like the moral uncertainty argument is regularly offered as a reason to ensure that human cells do not contribute to the brains of human-pig chimeras created via interspecies blastocyst complementation (see eg, refs 1 20 21).iii  Similarly, the broader literature on part-human chimeras generally concurs with the view that chimeras with humanised brains raise greater ethical concerns than other areas of chimera research, especially in cases where the host animal is closely related to humans^8 18^—concerns that are presumably also based on the uncertain moral status of these chimeric animals. The problem is that the moral uncertainty argument does not only apply to human-animal chimeras with humanised brains. As we show below, we also have good reason to view the moral status of non-chimeric pigs as highly uncertain.

## Moral uncertainty regarding non-chimeric pigs

It is difficult to assess the cognitive abilities of pigs. Since we cannot communicate with pigs directly, we are limited to drawing inferences from pig behaviour and physiology. Some research has been conducted into pig cognition, and while it is unclear exactly what inferences we should draw from the relevant findings, this research provides ample reason to take pigs’ cognitive abilities seriously. Pigs react to the emotional state of other pigs, suggesting a capacity for empathy. Pigs have been trained to comprehend a basic language combining symbols for actions and objects (eg, ‘fetch the frisbee’). Some (non-chimeric) pigs are able to manipulate a modified joystick to align an on-screen cursor with a specific target, which arguably suggests self-awareness and self-agency. Finally, pigs have been documented behaving in ways that suggest sophisticated forms of social cognition.^26^ This last category of research has revealed some particularly striking behaviours. Consider a study that tasked pairs of pigs with foraging together daily in a specially designed arena. The smaller pig learnt the location of the food in advance, whereas the larger pig did not. After multiple sessions the larger pig usually began to follow their pair-mate to the food, then push them aside to claim the meal for themselves. In other words, the larger pig appeared to realise their pair-mate knew something they did not, then used this knowledge to exploit their pair-mate. After further foraging sessions the smaller pig would not head to the food until their partner was distant, out of sight, or otherwise occupied, apparently manipulating their pair-mate in order to keep the meal for themselves.^27^

It is difficult to know exactly what to make of such findings. On the one hand, there is a risk we might err on the side of anthropomorphic bias and mistake behaviours grounded in relatively unsophisticated cognitive processes as evidence of a sophisticated, human-like mind. On the other hand, we might make the opposite mistake—one that Frans de Waal has labelled ‘anthropodenial’^28^—and mistakenly assume these behaviours are properly explained in terms of less sophisticated cognitive abilities than pigs actually possess. Until we work out how to strike the right balance, and pending further research into pigs’ cognitive abilities, the nature and sophistication of pigs’ cognitive abilities will remain an open question.

Not only are the cognitive abilities of pigs uncertain, so too is the moral salience of whatever cognitive abilities pigs actually do possess. There are many philosophical accounts of the grounds of moral status. Some accounts are unlikely to be met by non-chimeric pigs. For example, Insoo Hyun claims full moral status is conferred by human self-consciousness—understood as a kind of higher order mental awareness of one’s own mental experiences—which Hyun believes requires a facility for language unique to humans.^24^ Other plausible baselines for moral status are based on characteristics that might be shared by at least some non-human animals, such as sentience, agency, autonomy, self-awareness, rationality, moral agency and sociability. It seems likely that pigs would have a high degree of moral status on at least some of these accounts. Indeed, some plausible baselines for moral status—such as sentience—are relatively undemanding, and are almost certainly met by pigs. Until we work out which account of moral status to apply, the moral salience of pigs’ cognitive abilities will be uncertain *even if* we can determine what cognitive abilities pigs typically possess.

The moral uncertainty argument against growing human organs in human-pig chimeras and the moral uncertainty argument against farming pigs for food are not perfectly analogous. On the one hand, it is possible that human-pig chimeras would develop greater cognitive abilities than non-chimeric pigs due to the contribution of human cells to the chimeric pigs’ brains; accordingly, human-pig chimeras may be more likely to possess full moral status than regular pigs. In this respect, farming human-pig chimeras with partly humanised brains might be ethically riskier than farming non-chimeric pigs. But on the other hand, the creation of human-pig chimeras aims to satisfy a morally weighty goal—that is, to save lives—whereas farming pigs to produce meat serves only to satisfy some people’s culinary preferences. Satisfying culinary preferences is significantly less morally important than saving lives. In this respect, farming pigs for food is ethically riskier than farming human-pig chimeras to provide organs for transplant. Given this difference in the moral stakes, it seems that the moral uncertainty argument should weigh at least as strongly against the farming of non-chimeric pigs for meat as it weighs against the farming of human-pig chimeras for their organs.

It seems, then, that two widely held moral views are inconsistent with each other. On the one hand, the uncertain moral status of human-pig chimeras is thought to provide a significant moral consideration against pursuing such research, or at least in favour of moving forward cautiously (eg, by ensuring human cells do not make a meaningful contribution to chimeric pigs’ brains). On the other hand, the uncertain moral status of non-chimeric pigs is rarely recognised as a compelling reason to stop farming them. The question, then, is how this tension ought to be resolved.

## Resolving the tension

Many people endorse a precautionary approach to human-pig chimera research because of concern about the possibility that such animals have sufficient moral status that killing them is a serious moral wrong. (For simplicity, we refer to quality as ‘full moral status.’) This might be called the moral status precautionary principle (MSPP).

*Moral status precautionary principle*: A course of action should not be pursued if there is a reasonable fear that the course of action will cause serious harm to beings of full moral status, even if there is no conclusive evidence that the beings will actually have full moral status.

Like other versions of the precautionary principle, the MSPP faces a distinctive challenge.^29 30^ The challenge runs as follows. It is possible to imagine scenarios where both pursuing a course of action and *failing* to pursue the same course of action will trigger the precautionary principle (in the case of the MSPP, by risking serious harm to beings of full moral status). In such contexts, the precautionary principle leads to the paradoxical conclusion that it would be wrong to pursue *and* to fail to pursue a particular course of action. For this reason, some philosophers hold that precautionary principles are incoherent.

This objection is not necessarily fatal to the MSPP. Proponents of other kinds of precautionary principle have argued that this objection can be met by more carefully specifying the conditions under which the precautionary principle applies—for example, by specifying that the principle applies only to threats of a particular magnitude and probability, or by developing mechanisms to ensure precautionary measures are proportional to the size of the threat.^31 32^ A similar response might be open to proponents of the MSPP.

Alternatively, one could reject the MSPP and endorse the less demanding moral status no alternative principle (MSNAP), which would allow the farming of human-pig chimeras *only* if there are no alternative means of addressing the organ shortage:

*Moral status no alternative principle*: A course of action may be pursued if there is a reasonable fear that the course of action will cause serious harm to beings of full moral status, *only* if there is no alternative course of action that would achieve the same benefit without any risk of serious harm to beings of full moral status.^iv^

Neither the MSPP nor the MSNAP necessarily captures the complete moral picture. The underlying moral question at play is about how we should approach decision-making when we are unsure about moral facts salient to a decision. The MSPP and MSNAP are not the only, nor necessarily the best, approaches one could take.^v^ However, we think they are nonetheless a good approximation of the kinds of intuitions that underlie many people’s reluctance to inflict serious harms on human-pig chimeras with humanised brains. The MSPP and the MSNAP will therefore suffice for the purposes of this paper.

Both the MSPP and the MSNAP are consistent with the view that we should require confidence that human-pig chimeras would lack full moral status before using them to grow transplantable human organs. But if one accepts the MSPP or MSNAP in relation to human-pig chimeras, then one also ought to accept these principles in relation to regular, non-chimeric pigs. Given that there is a plausible alternative to farming pigs for food (vegetarianism), this would seem to entail that we renounce the farming of pigs for food. Conversely, if one accepts the farming of non-chimeric pigs for food (and therefore rejects the MSPP/MSNAP’s application to regular pigs), then one should presumably also reject the MSPP/MSNAP’s application to human-pig chimeras. How, then, should we bring our views into alignment?

One approach is to reject the precautionary principle’s application to either human-pig chimeras or non-chimeric pigs. One might hold that the cognitive capacities associated with full moral status are fleetingly unlikely to emerge in anything other than a *fully* human brain that is housed in a *fully* human body. Indeed, some commentators have made precisely this argument to attempt to defuse concerns about human-animal chimeras’ moral status.^24 33^ This view does not give rise to the kind of tension described in this paper. Rather than singling out human-pig chimeras for special moral concern, it denies that either human-pig chimeras or non-chimeric pigs could plausibly possess a substantial degree of moral status.

Is this a satisfactory way to harmonise our views on the moral status of pigs and human-pig chimeras? It is at least superficially attractive, not least because it preserves deeply ingrained intuitions that our current uses of non-human animals are ethically appropriate. However, we may have good reason to treat these intuitions with suspicion. There is a burgeoning body of literature that suggests our estimation of non-human animals’ moral status is highly flexible and, importantly, shaped by whether or not we classify a particular animal as food.^34^ Consider, for example, a study in which students were asked to sample a certain kind of food (either beef jerky or cashew nuts) and then—for what appeared to be an unrelated project—asked their opinions of the cognitive capacity and moral status of a range of animal species. Those who were assigned to the beef jerky group were more inclined to deny that food animals are due moral consideration than those students assigned to the cashew nut group.^35^ Our willingness to eat pigs might not follow from a considered judgement that pigs lack morally relevant cognitive capacities; instead, our judgement that pigs lack morally relevant cognitive capacities might be distorted by psychological mechanisms we use to render our consumption of them more palatable.

A second possible solution is to apply the MSPP/MSNAP to both human-pig chimeras and non-chimeric pigs. If we think it is appropriate to take precautions against harming human-pig chimeras on the chance they have full moral status, then perhaps we should also be prepared to take precautions against harming regular pigs on the chance *they* possess full moral status. This would require us to renounce the farming of pigs for food unless and until we can be confident that pigs lack more than minimal moral status.

On balance, we think this second approach is more promising than the first. We have good reason to be wary of our intuitions regarding the moral status of non-chimeric pigs, for (as outlined above) our intuitions may be shaped by a powerful bias against attributing sophisticated cognitive abilities to pigs or according them moral status. In comparison, our moral views on human-pig chimeras—a new type of creature which would presumably not be regarded as food, and which is not yet enmeshed in any culturally valued practices—are presumably not be subject to the same bias. Arguably, then, we have good reason to view the moral status of both chimeric *and* non-chimeric pigs as uncertain, and to apply the MSPP/MSNAP to both kinds of beings.

There is one further possibility. Perhaps both sets of attitudes require revision. We might be liable to overestimate the moral status of human-pig chimeras (because they are part human) *and also* liable to underestimate the moral status of regular pigs (because they are not). Perhaps concerns about the moral status of both human-pig chimeras should be considered somewhat less pressing than is commonly assumed, whereas concerns about the moral status of non-chimeric pigs should be considered somewhat more pressing than is commonly assumed.

In any case, this paper’s primary aim has been to highlight an important tension between two widely held moral views, not to definitely resolve that tension. Exactly how we should realign our views is open for further debate; we have mainly sought to highlight a tension in existing ethical thought that we think needs to be resolved.

## Possible objections

We can see two potential objections to our argument.^vi^ These objections attempt to show that *only* human-pig chimeras (and not regular pigs) have uncertain moral status—in which case one could consistently hold that farming human-pig chimeras raises serious moral concerns, whereas farming regular pigs does not. In this section we show how each objection can be met.

The first objection runs as follows. We have argued that those who apply the MSPP/MSNAP to the farming of human-pig chimeras should also apply the same principle to the farming of non-chimeric pigs. But human-pig chimera research has a unique feature: it raises the possibility of raising the animals’ cognitive abilities above the level of regular pigs. On this view, there is no inconsistency in holding that the moral status of human-pig chimeras is uncertainwhile also holding that regular pigs are certain to possess no more than a negligible degree of moral status. Perhaps the reason why many people accept the farming of non-chimeric pigs (but nonetheless find the farming of human-pig chimeras morally troubling) is because they are already certain that non-chimeric pigs lack sufficient moral status that killing them is a serious moral wrong.

We do not think this is a tenable view. To defend it, one would have to pinpoint some morally relevant feature(s) that human-pig chimeras could plausibly develop but that non-chimeric pigs certainly lack. This would be a very difficult task. As highlighted earlier, there is both significant philosophical disagreement regarding the grounds of moral status and significant scientific disagreement regarding the nature and sophistication of pig cognition. It is philosophically difficult—potentially far more difficult than many people realise—to pinpoint some set of cognitive capacities that can do the necessary moral work. Moreover, there is considerable philosophical debate and argument that advances the view that non-chimeric pigs *do *possess capacities that merit moral consideration.^36 37^ Given this debate, it appears difficult to defend a view that pigs *certainly* lack moral status. We suggest that anyone familiar with debates about animal ethics ought to admit there is at least a non-trivial possibility that (non-chimeric) pigs have cognitive capacities meriting moral consideration.

Perhaps we can dissolve the moral tension by linking moral status to something other than a being’s cognitive capacities. Perhaps what motivates concerns about chimeric animals’ moral status is not that they possess sophisticated cognitive capacities, but that they are partly *human.* In other words, one might think that (full) moral status depends on what species one belongs to rather than the cognitive capacities one happens to possess. In line with this view, the moral status of human-pig chimeras might be uncertain because they are neither fully human nor fully non-human. The moral status of regular pigs, however, is easy to settle; regular pigs are entirely non-human and therefore certainly lack (full) moral status. Accordingly, those who think that *humanness* is what confers (full) moral status might deny that the MSPP/MSNAP weighs against the farming and consumption of regular, non-chimeric pigs.vii

There are two reasons we do not think this objection succeeds. First, the idea that *humanness* confers moral status seems to play little role in current debates about the generation of human organs in chimeric animals. If concerns about interspecies blastocyst complementation *were* based on the blurring of species boundaries, they would presumably apply regardless of whether the chimeric animal develops unexpected cognitive abilities. However, the literature surveyed at the beginning of this paper suggests that interspecies blastocyst complementation is controversial primarily because of the risk that human cells would contribute to the brain and thereby affect the chimeric animal’s cognition. Indeed, one common suggestion made throughout this literature—that we should limit the degree of chimerism in (some) chimeric animals’ brains—would make little sense if one thought that introducing human cells per se renders chimeric animals’ moral status uncertain. A pig with a human pancreas is not entirely non-human, even if human cells are absent from the rest of its body. However, few commentators have suggested that possessing a human pancreas could increase the moral status of a pig beyond that of its regular, non-chimeric counterparts. In much the same vein, few people believe that humans who receive a porcine heart valve replacement risk diluting their own moral status.

But more importantly, there are good philosophical reasons to doubt that species membership is relevant to moral status. Consider the following thought experiment from David DeGrazia. DeGrazia describes the recent discovery of what is thought to be a distinct hominid species—*Homo floresiensis*—that seem to have developed and used sophisticated tools. Using *H. floresiensis* as an example, DeGrazia asks whether we should ascribe (full) moral status to non-human hominid species that possess cognitive abilities similar to those of present-day humans. The answer, presumably, is yes. But if this is the case, species membership per se must be irrelevant to moral status:

[T]o deny that these hominids—or beings like them and like us—lack(ed) moral status merely because of species difference is the height of bigotry. Even if biology were so morally important—and it isn’t—there would be no reason to think that *species* markers are so important to moral status. After all, these recent hominids… were members of our genus, *Homo.* And all members of the genuses *Australopithecus* and *Paranthropus* were, like members of *Homo,* hominids, another biological grouping. And, of course, all of these and many other animals are apes, primates, mammals, vertebrates, and so on. To single out species as the unique biological basis for moral status is as silly intellectually as it is self-serving for those in whom species prejudice operates strongly.^38^

If we are to ascribe moral status to some beings and not to others, we will need to appeal to some feature other than species membership. The answer, presumably, lies in the mental life of the being in question. But if we are to ascribe moral status on the basis of a being’s cognitive capacities, we will need to confront the possibility that beings other than humans—including pigs—have the requisite set of morally relevant characteristics.viii

## Conclusion

We have argued that two common moral views are in tension with each other. On the one hand, it is widely believed that the creation of human-pig chimeras with partly humanised brains would raise weighty moral concerns, largely because the moral status of such animals would be uncertain. On the other hand, it is widely believed that our current use of non-chimeric pigs as livestock is morally benign. In both cases, however, there are grounds for moral uncertainty. First, we are faced with difficult questions regarding what set of cognitive capacities are morally relevant. Second, we are faced with the difficulty of testing for these capacities in creatures that look very different to us, that behave in very different ways and that we cannot communicate with directly. It is a problem, then, that currently only the farming of human-pig chimeras currently seems to attract serious moral concern. Until we resolve this inconsistency our treatment of human-pig chimeras and/or non-chimeric pigs will almost certainly rest on a serious moral mistake.

## Footnotes

i Using human-pig chimeras to generate human organs also raises concerns about animal welfare, the ‘unnaturalness’ of crossing species boundaries and the risk of transmitting novel infections with the transplantable organs.^21^ These issues fall outside the scope of this paper.

ii This is consistent with the view—defended by Harry Bradshaw,^39^ Simon Knutsson and Christian Munthe,^40^ and Jeff Sebo^41^—that if we are uncertain of some being’s moral status, we ought to treat them as having at least partial moral status.

iii This leaves open the question of whether it is ethically permissible to create chimeric animals with humanised brains to study and/or develop new treatments for neurodegenerative disorders (see eg, ref 42). It is obviously impossible to conduct such forms of research without creating chimeric animals with humanised brains.

iv The MSNAP resembles a well-established principle in animal ethics: that it is permissible to conduct harmful forms of animal research *only* if there is no alternative way to answer the scientific question.^43^

v For a general discussion of decision-making under moral uncertainty, see ref 44. For a broader overview of different ways one could respond to uncertainty regarding moral status, see ref 41.

vi We thank two anonymous peer reviewers for raising these objections and pressing us to respond.

vii One might still think that harming pigs might coarsen human attitudes towards other humans, display a lack of virtue, or be wrong for some other reason. But denying that pigs have (full) moral status voids the most central criticism against practices that harm pigs—which is that these practices are wrong by virtue of the harms inflicted on the pigs themselves.

viii Even if one agrees that the moral uncertainty argument leads to the right conclusions regarding human-pig chimeras and non-chimeric pigs, one might worry that this argument has unpalatable implications for the abortion debate. Like the farming of human-pig chimeras and regular pigs, abortion involves killing a being that at least some moral views grant full moral status.  We do not think our argument has problematic implications for the ethics of abortion. Of the two moral principles we have been considering, only the MSPP *might* weigh against abortion. The MSPP would rule out abortion if there is reasonable fear that, by terminating a pregnancy, one would inflict serious harm on a being of full moral status. Some philosophers have offered arguments to this effect,^45^ but whether such arguments succeed is beyond the scope of this paper.  The MSNAP applies only if there is some alternative course of action that would achieve one’s ends without risking serious harm to beings of full moral status. In the case of abortion, no such alternative is available; one cannot achieve the goal of respecting a woman’s wish not to continue a pregnancy without ending the life of the fetus. Accordingly, the MSNAP does not support any specific position on the ethics of abortion.
